# Late neurological consequences of SARS-CoV-2 infection: New challenges for the neurologist

**DOI:** 10.3389/fnins.2023.1004957

**Published:** 2023-02-09

**Authors:** Agnieszka Korchut, Konrad Rejdak

**Affiliations:** Department of Neurology, Medical University of Lublin, Lublin, Poland

**Keywords:** COVID-19, SARS-CoV-2, long haul, neurological manifestation, neurological complication, neuro-COVID-19, post-COVID-19

## Abstract

**Objective:**

In this study, a systematic review of the literature was performed to study the frequency of neurological symptoms and diseases in adult patients with COVID-19 that may be late consequences of SARS-CoV-2 infection.

**Methods:**

Relevant studies were identified through electronic explorations of Scopus, PubMed, and Google Scholar. We followed PRISMA guidelines. Data were collected from studies where the diagnosis of COVID-19 was confirmed and its late neurological consequences occurred at least 4 weeks after initial SARS-CoV-2 infection. Review articles were excluded from the study. Neurological manifestations were stratified based on frequency (above 5, 10, and 20%), where the number of studies and sample size were significant.

**Results:**

A total of 497 articles were identified for eligible content. This article provides relevant information from 45 studies involving 9,746 patients. Fatigue, cognitive problems, and smell and taste dysfunctions were the most frequently reported long-term neurological symptoms in patients with COVID-19. Other common neurological issues were paresthesia, headache, and dizziness.

**Conclusion:**

On a global scale of patients affected with COVID-19, prolonged neurological problems have become increasingly recognized and concerning. Our review might be an additional source of knowledge about potential long-term neurological impacts.

## Introduction

There is growing evidence indicating that neurological manifestations occur in patients as sequelae of COVID-19 (Misra et al., 2021). Approximately one-third of positive patients develop neurological and neuropsychiatric symptoms (Rudroff et al., 2020).

SARS-CoV-2 neurotropism has been increasingly recognized by its imaging and clinical manifestations from severe (encephalitis) to mild (hyposmia) in the literature. The neurological symptoms profile associated with COVID-19 covers symptoms of the central nervous system, peripheral nervous system, and neuromuscular disorders. The impact of SARS-CoV-2 on the nervous system is associated with the following issues: olfactory and taste disorders, Guillain–Barre syndrome (GBS), encephalopathy, neurological inflammation (myelitis, encephalitis, and meningitis), cerebrovascular diseases, seizures, cognitive impairment, myalgia, non-specific symptoms such as headache, dizziness, and fatigue, or neuropsychiatric symptoms such as anxiety, depression, psychosis, and sleep disorder (Divani et al., 2020; Collantes et al., 2021; Roy et al., 2021; Yassin et al., 2021). Most infected people develop mild to moderate illness and recover without requiring hospitalization, while others must be hospitalized.

Neurological symptoms are not necessarily correlated with the severity of COVID-19 infection, implying that different mechanisms or timing of mechanisms may be involved (Rogers et al., 2021).

These symptoms can appear in three disease periods, such as acute (parainfectious), post (postinfectious), and late infections (long-term sequelae). Datta et al. (2020) presented a theoretical timeframe for periods of SARS-CoV-2 infection: acute infection (from the onset of symptoms up to 2 weeks), post-acute infection (2 weeks after initial infection), and late sequelae (4 weeks after initial infection) (Datta et al., 2020). According to current knowledge, concerning the duration of neurological manifestation from COVID-19 symptoms onset, neurological issues can be placed in a timeframe.

Regarding cerebrovascular diseases, most manifestations occur within 21 days from COVID-19 onset, and stroke was rarely the first manifestation (Vogrig et al., 2021).

In the literature, neurological inflammation related to COVID-19 is observed as para- or postinfectious disease (Paterson et al., 2020). On average, encephalitis occurred 14.5 days after the diagnosis of COVID-19 infection (range = 10.8–18.2 days) (Siow et al., 2021).

Cases of Guillain–Barre syndrome in patients with COVID-19 have been described as a parainfectious disease (Romoli et al., 2020) or a postinfectious disease with a 2-week interval between SARS-CoV-2 and GBS infection (Palaiodimou et al., 2021).

It is known that severe acute respiratory syndrome coronavirus (SARS) and Middle East respiratory syndrome coronavirus (MERS) may have prolonged neurological impact (Ngai et al., 2010; Hosseiny et al., 2020). Emerging evidence suggests that the neuroinvasive nature of COVID-19 may be the driving force behind late neurological complications.

An increasing number of patients with COVID-19 continue to experience symptoms for months, even after recovering from mild cases of COVID-19 such as muscle pain, dizziness, headaches, fatigue, and anosmia (Wijeratne and Crewther, 2020), as well as signs and symptoms involving cognitive functions (Baig, 2020). Qin et al. (2021) found that the patients with mild- and severe-type COVID-19 with no specific neurological manifestations or obvious lesions on the conventional MRI, although recovered from pneumonia, still exhibited brain microstructure changes and a decrease in cerebral blood flow after a 3-month follow-up (Qin et al., 2021). For healthcare professionals and scientists, the prolonged neurological impact is a new challenge. In the literature, we can find different nomenclature for this phenomenon as Chronic COVID syndrome (CCS) (Baig, 2020), Post-COVID-19 Neurological Syndrome (PCNS) (Wijeratne and Crewther, 2020), Long COVID, and Long hauler COVID (Mendelson et al., 2020).

This review focused on the neurological symptoms and diseases that may be late consequences of SARS-CoV-2 infection.

## Methods

The databases Scopus, PubMed, and Google Scholar were reviewed before 4 June 2022. An individual database search strategy was adopted with the following variations of keywords: (“COVID-19” OR “COVID19” OR “neuro-covid 19” OR “Sars Cov-2” OR “coronavirus”) AND (“long term” OR “chronic” OR “long haul^*^” OR “post-covid^*^” OR “post covid^*^” OR “long covid^*^”) AND (“neurological manifestation” OR “neurological complication” OR “neurolog^*^”). The first step was the title screening; the second was the abstract screening, and the third step was a full-text review of the relevant information. We followed PRISMA guidelines.

### The inclusion criteria

The criteria for inclusion in the publication review were as follows:

- English and German language publications, which reported post-COVID-19 neurological issues among the adult population (subjective and/or objective) and
- data were collected from studies where all the patients were confirmed with positive SARS-CoV-2 PCR or antibody test.

The time criteria for identifying neurological issues were at least 4 weeks after the initial SARS-CoV-2 infection.

### The exclusion criteria

We excluded review articles and publications that relied on the analysis of neurological symptoms associated with previous outbreaks (SARS in 2003; MERS in 2012). In addition, neuropsychiatric symptoms such as anxiety, depression, psychosis, and sleep disturbances were not included. Database searches were combined and duplicates were removed.

The following variables were extracted from included studies: first author, type, year and source of publication, research country, sample size, neurological issues, methods, and time when symptoms were identified. In addition, attention was paid to the severity of the COVID-19 disease course, whose severity was measured by hospitalization status without distinction between hospitalization in the intensive care unit. Descriptive analyses were applied. The incidence of neurological issues was presented as numbers and percentages. If in the analyzed article there were more follow-up visits within the time criterion of our study, the most numerous study group was selected. However, if there was the same sample size at the follow-up visits, the last visit was selected.

### Categorization of neurological symptoms

To summarize the neurological issues, we had to define a cluster of cognitive problems. Such a cluster was defined as any subjective reports of concentration, memory and attention difficulty, perceived “brain fog”, disorientation/confusion, word-finding difficulty, inability to effectively multitask, and measurable cognitive impairment confirmed by a test. “Frequent,” “more frequent,” and “the most frequent” neurological consequences were defined to have a frequency above 5, 10, and 20%, respectively, and were reported in at least five different studies including at least 1,000 of all patients studied. The term “possible significant neurological consequences” was used if the frequency of symptoms was reported above 15% in at least three publications, with a total study group of at least 300.

## Results

### Included types of publications—Short characteristic

**Figure 1:**
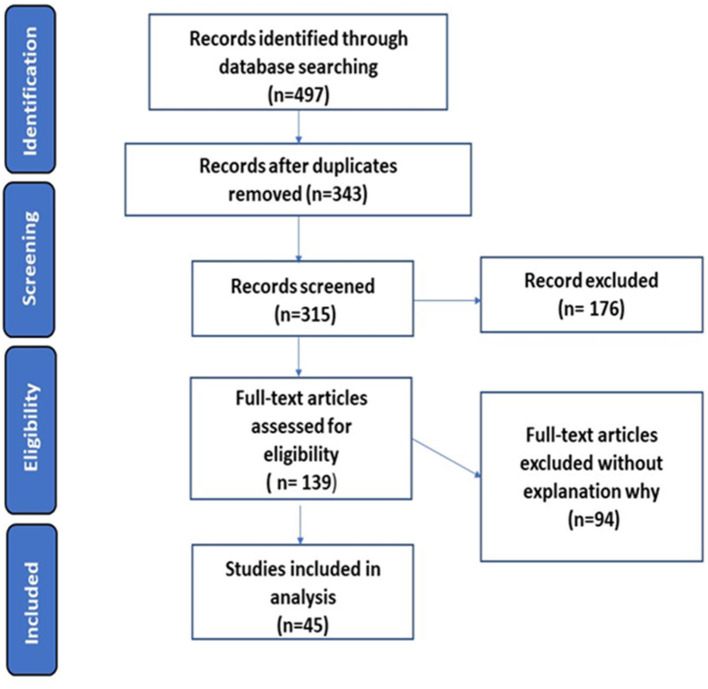
PRISMA flow diagram.

A total of 497 articles were identified for eligible content. After excluding duplicates and screening titles and abstracts, which did not meet inclusion criteria, 139 full-text publications were assessed. From full-text publications, 94 were excluded due to the non-relevance of the investigated topic. Our review included data from 45 articles: retrospective studies (2), prospective studies (21), case reports/series (15), and cross-sectional studies (7). PRISMA flow diagram is presented in Figure 1. Features of studies included in our review are summarized in Table 1.

**Table 1:** Summarized features of the included studies.

			**Characteristics of studies**			
**References**	**Type of publication**	**Research country**	**Sample size (** ***N*** **)**	**Hospitalization status (H** ^*^ **/NH** ^**^ **/M** ^***^ **)**	**Neurological issues [*****n*** **(%)]**	**Time to identified symptoms, methods**
Romero-Duarte et al. (2021)	Retrospective study	Spain	*N*: 797	H	Persistent anosmia or dysgeusia [57 (7%)] Muscular debility acquired in ICU [25 (3%)] Headache [42 (5%)] Paresthesia [27 (3%)] Movement disturbances [27 (3%)] Disorientation or confusion [21 (3%)] Vertigo [15 (2%)]	After 6 months hospital discharge, collected through clinical histories and primary care reports
Vanichkachorn et al. (2021)	Case series	USA	*N*: 100 *N*: 75	M, NH	Fatigue [80 (80%)] Headache [20 (20%)] Dizziness [19 (19%)] Paresthesia [17 (17%)] Persistent altered taste/smell [9 (9%)] Cognitive impairment [45 (45%)]	At least 4 weeks after a positive SARS-CoV-2 polymerase chain reaction (PCR) test and/or symptomatic start of confirmed SARS-CoV-2 infection, face-to-face visits and/or virtually by using either video telemedicine or telephone interactions.
Bozzali et al. (2021)	Case report	Italy	*N*: 1	NH	Focal seizures with impaired awareness	Two months after acute phase of SARS-CoV-2 infection MRI CSF analysis Blood test [18F]FDG positron emission tomography (for the exclusion of other causes)
Orr et al. (2021)	Retrospective study	Italy	*N*: 74	M	Fatigue [59 (80%)] Headache [40 (54%)] Cognitive impairment [44 (59%)] Dizziness [25 (34%)] Paresthesia [26 (35%)] Loss of taste [21 (28%)] Loss of smell [24 (32%)] Myalgia [44 (59%)]	Three follow-up visits (time relative to negative swab of SARS-CoV-2) at least a month, at least 2 months, at least 3 months, survey
			*N*: 154	M	Fatigue [121 (79%)] Headache [76 (49%)] Cognitive impairment [73 (47%)] Dizziness [33 (21%)] Paresthesia [48 (31%)] Loss of taste [39 (25%)] Loss of smell [42 (27%)] Myalgia [81 (53%)]	
			*N*: 152	M	Fatigue [113 (74%)] Headache [71 (47%)] Cognitive impairment [74 (49%)] Dizziness [34 (22%)] Paresthesia [53 (35%)] Loss of taste [41 (27%)] Loss of smell [47 (31%)] Myalgia [93 (61%)]	
Miskowiak et al. (2021)	Prospective study	Denmark	*N*: 29	H	Cognitive impairment [17 (60%)]	3-4 months after discharge, Cognitive Impairment in Psychiatry Danish Version (SCIP-D), Trail Making Test-Part B (TMT-B), Cognitive Failures Questionnaire (CFQ)
Graham et al. (2021)	Prospective study	USA	*N*: 50	NH	Headache [32 (16%)] Brain fog [41 (20.5%)] Dizziness [20 (10%)] Numbness/tingling [29 (14.5%)] Myalgia [30 (15%)] Dysgeusia [32 (16%)] Anosmia [37 (18.5%)] Blurred vision [9 (4.5%)] Fatigue [42 (21%)] Abnormal movement [2 (1%)] Sensory dysfunction [3 (1.5%)] Cranial nerve dysfunction [5 (2.5%)] Dysarthria [2 (1%)] Dysphagia [1 (0.5%)] Short-term memory deficit1 [5 (2.5%)] Attention deficit [12 (6%)] Motor dysfunction [3 (1.5%)] Gait dysfunction [3 (1.5%)] Cerebellar dysfunction [1 (0.5%)]	More than 6 weeks from symptoms onset Evaluated for in-person visits and telemedicine 4-item recall Serial 7s
Raahimi et al. (2021)	Case report	UK	*N*: 1	H	GBS	53 days after having SARS-CoV-2 infection CSF test NCS
Carfi et al. (2020)	Case series	Italy	*N*: 143	H	Anosmia [ 21 (15 %)] Headache [ 14 (10%)] Vertigo [7 (5%)]	Mean of 60.3 (SD, 13.6) days after onset of the first COVID-19 symptom Medical assessment with detailed history and physical examination
Carvalho-Schneider et al. (2020)	Prospective study	France	*N*: 150 *N*: 116	M NH	Anosmia/ageusia [40 (27.8%)]	Two follow-up visits: 1 month and 2 months after COVID-19 symptoms onset, data collected by phone call
			*N*: 130 *N*: 101	M NH	Anosmia/ageusia [29 (22.7%)]	
Kayaaslan et al. (2021)	Prospective study	Turkey	*N*: 1,007 *N*: 591	M NH	Concentrations and memory deficit [163 (16.2%)] Headache [57 (5.7%)] Loss of smell [31 (3.1%)] Loss of taste [21 (2.1%)]	At least 3 months after SARS-CoV-2 infection, survey
Anaya et al. (2021)	Case series	Colombia	*N*: 100 *N*: 35	M NH	Back pain [55 (55%)] Headache [45 (45%)] Paresthesia [38 (38%)] Attention disorders [36 (36%)] Memory disorders [36 (36%)] Anosmia [11 (11%)] Dizziness [31 (31%)] Seizures [1 (1%)]	The median of post-COVID-19 time was 219 days (IQR: 143–258), survey	
Garrigues et al. (2020)	Case series	France	*N*: 120	H	Ageusia [13 (10.8%)] Anosmia [16 (13.3%)] Attention disorder [32 (26.7%)] Memory loss [41 (34.2%)]	At least 100 days after admission for COVID-19, questionnaire
Santis et al. (2020)	Prospective study	Spain	*N*: 108	NH	Headache [10 (9.3%)] Anosmia [10 (9.3%)] Dysgeusia [5 (5.6%)] Loss of memory [2 (1.9%)] Difficulty of concentrating [2 (1.9%)]	12 weeks after acute phase of SARS-CoV-2 infection, medical history and examination
Woo et al. (2020)	Cross-sectional study	Germany	*N*: 18 *N*: 7	M NH	Mild cognitive deficits [14 (78%)] Attention deficits [9 (50%)] Concentration deficits [8 (44.4%)] Short-term memory deficits [8 (44.4%)] Troubles in finding words [5 (27.8%)]	At least 20 days after recovery from SARS-CoV-2 infection, TICS-M (Modified Telephone Interview for Cognitive Status)
Leth et al. (2021)	Prospective study	Denmark	*N*: 49	H	Difficulties concentrating [19 (39%)] Impaired OMC test (*N*: 38) [8 (21%)] Paresthesia [ 8 (16%)] Headache [12 (24%)] Smell impairment1 [7 (35%)] Taste impairment [16 (33%)] *N*: 49 (H) Difficulties concentrating [22 (45%)] Impaired OMC test (*N*: 38) [4 (11%)] Paresthesia [13 (27%)] Headache [13 (27%)] Smell impairment [13 (27%)] Taste impairment [15 (31%)]	Two follow up visits: 6 and 12 weeks after discharge, medical history, OMC test (orientation, memory, and concentration)
Sykes et al. (2021)	Prospective study	UK	*N*: 78	H	Fatigue [26 (33.3%)] Myalgia [33 (42.3%)] Memory impairment [24 (30.8%)] Attention deficit [16 (20.5%)] Anosmia [8 (10.2%)] Cognitive impairment [4 (5.1%)] Taste deficiency [6 (7.7%)]	At least 101 days after discharge (101–125) Clinical assessment
Halpin et al. (2021)	Cross-sectional study	UK	*N*: 100	H	New or worsened concentration problem [22 (22%)] New or worsened short-term memory problem [18 (18%)]	4–8 weeks after discharge, specialist telephone screening tool
Iqbal et al. (2021)	Cross-sectional study	Pakistan	*N*: 158	M	Loss of smell and taste [75 (47.5%)] Headache [57 (36.1%)] Brain fog [30 (19.0%)] Blurred vision [30 (19.0%)] Stroke [1 (6%)]	At least 20 days after recovery from COVID-19, questionnaire
Huang et al. (2021)	Prospective study	China	*N*: 1655	H	Fatigue or muscle weakness [1,038 (63%)] Smell disorder [176 (11%)] Taste disorder [120 (7%)] Dizziness [101 (6%)] Headache [33 (2%)]	Six months after discharge, series of questionnaires Physical examinations
Kanberg et al. (2021)	Prospective study	Sweden	*N*: 97	M	Fatigue [40 (41%)] Brain fog [29 (30%)] Changes in cognition [25 (26%)] Hyposmia [4 (4%)] Dysgeusia [5 (5%)]	Six months follow up, questionnaires
Stuby et al. (2021)	Case report	Switzerland	*N*: 1	H	Guillain–Barré syndrome (GBS)	1 month after SARS-CoV-2 infection NCS CSF analysis
Aasfara et al. (2021)	Case report	Marocco	*N*: 1	NH	Guillain–Barré syndrome (GBS) associated to a vestibulocochlear neuritis SARS-CoV-2 positive 6 weeks before (GBS)	6 weeks after a positive SARS-CoV-2 test, NCS, CSF analysis, audiometry and videonystagmography
Alemanno et al. (2021)	Prospective study	Italy	*N*: 56	H	Cognitive deficit [41 (73%)]	1 month after home discharge MoCA
Zhang et al. (2021)	Prospective study	China	*N*: 2,433	H	Fatigue [696 (27.7%)] Dizziness [82 (3.3%)] Headache [57 (2.3%)] Impaired sense of smell [32 (1.3%)]	At 1-year follow-up visit, questionnaires
Papri et al. (2021)	Case report	Bangladesh	*N*: 1	H	Guillain–Barré syndrome (GBS)	Six weeks after SARS-CoV-2 infection, NCS
Hellmuth et al. (2021)	Prospective study and two cases	USA	*N*: 14	NH	Cognitive deficits (symptoms were present for at least a median 98 days onset COVID-19)	At least a median 98 days after the onset of SARS-CoV-2 infection, medical interview
			*N*: 2	NH	Cognitive deficit	72 and 149 days after the onset of SARS-CoV-2 infection, California Verbal Learning Test-3 (16-word) WAIS Wechsler Adult Intelligence Scale IV Digital Span
Albu et al. (2021a)	Cross-sectional study	Spain			*N*: 30 (M) 7 (NH) Cognitive impairment [19 (63.3%)] CIP/CIM: Critical illness polyneuropathy/myopathy [7 (23.3%)]	3 months after acute phase of SARS-CoV-2 infection, Benton Temporal Orientation Test, Wechsler Adult Intelligence Scale III, Rey Auditory Verbal Learning Test, PMR task (a Spanish version of the FAS letter fluency task)
Alvare et al. (2021)	Case report	USA			*N*: 1 (H) Extended neuralgic amyotrophy syndrome	Three weeks after discharge EMG, NCSs, and MRI.
Nakamura et al. (2021)	Case report	Japan			*N*: 1 (H) Restless legs syndrome variant	Several weeks after discharge, face-to-face interview and physical examination, colonoscopy, blood test
Poletti et al. (2021)	Cross-sectional study	Italy	*N*: 92 *N*: 122 *N*: 98	M M M	Cognitive impairment [73 (79%)] Cognitive impairment [92 (75%)] Cognitive impairment [67 (68%)]	Three follow up visits: 1-month and 2 and 3- months after discharge, Brief Assessment of Cognition in Schizophrenia (BACS), hospitalized/non hospitalized
Zhu et al. (2021)	Prospective study	China	*N*: 95	H	Hyposmia [22 (23.2%)]	At least 16 weeks after the onset of SARS-CoV-2 infection, Hyposmia Rating Scale (HRS), Brief Smell Identification Test for Chinese (B-SITC)
Boesl et al. (2021)	Prospective study	Germany	*N*: 100 *N*: 89	M NH	Cognitive impairment [72 (72%)] Fatigue [67 (67%)] Headache [36 (36%)] Hyposmia [36 (36%)] Myalgia [21 (21%)] Vertigo [20 (20%)] Limb pain [9 (9%)]	At least 12 weeks after acute phase of SARS-CoV-2 infection, questionnaires, Montreal Cognitive Assessment Scale (MoCA)
Ahmad and Salih (2021)	Case report	Iraq	*N*: 1	NH	Transverse myelitis	Two weeks after recovery from COVID-19, brain and cervical magnetic resonance imaging (MRI) CSF analysis
Frontera et al. (2021)	Prospective study	USA	*N*: 111	H	Cognitive impairment [50 (45%)] (without neurological complications during acute phase of SARS-CoV-2 infection)	Six months after discharge, Telephone MOCA (Montreal Cognitive Assessment)
			*N*: 90	H	Cognitive impairment [45 (50%)] (with neurological complications during acute phase of SARS-CoV-2 infection)	
Pistarini et al. (2021)	Cross-sectional study	Italy			*N*: 20 (H) Cognitive deficit [14 (70%)] (MoCA) [1 (5%)] (MMSE)	One month after SARS-CoV-2 infection, MMSE, MoCA
Park et al. (2021)	Case report	USA	*N*: 1	H	Focal seizures with impaired awareness	6 weeks after negative of SARS-CoV-2 test MRI CSF analysis Blood test EEG
Carroll et al. (2020)	Case report	USA	*N*: 1	H	Refractory status epilepticus	6 weeks after initial infection with COVID-19 MRI CSF analysis Blood test EEG
Albu et al. (2021b)	Prospective study	Spain	*N*: 40	M	Cognitive complains [15 (37.5%)] Fatigue [35 (87.5%)]	Over 3 months after initial infection with COVID-19 Benton Temporal Orientation Test Wechsler Adult Intelligence Scale III Rey Auditory Verbal Learning Test PMR task (a Spanish version of the FAS letter fluency task)
			*N*: 32	M	Cognitive deficit in tests [23 (72.2%)]	
Pilotto and Cristillo (2021)	Prospective study	Italy	*N*: 165	H	Fatigue [56 (33.9%)] Memory/concentration complaints [52 (31.5%)] Myalgia [50 (30.3%)] Blurring/loss of vision [32 (19.5%)] Paresthesia [31 (18.8%)] Hyposmia/hypogeusia [27 (16.4%)] Urinary dysfunction [23 (13.9%)] Confusion [22 (13.3%)] Hypotension [20 (12.2%)] Gait disturbance [18 (10.9%)] Abnormal movements [17 (10.3%)] Headache [16 (9.7%)] Postural instability or falls [14 (8.5%)] Swallowing difficulties [10 (6.1%)]	At 6-month follow-up visits, questionnaire Neurological examination NCS MoCA
			*N*: 105	H	Sensor-motor polyneuropathy [ 2 (2%)] Cognitive impairment in test [17 (17%)] Enhanced physiological tremor [15 (15%)] Dysgeusia/hyposmia [19 (19%)]	
Garg et al. (2021)	Case report	USA	*N*: 1	H	Functional movement disorders - abnormal repetitive movement of the head	2 months after acute phase of SARS-CoV-2 infection, MRI, EEG
Rivera-Izquierdo et al. (2022)	Prospective study	Spain	*N*: 453	H	Fatigue [37 (8.2%)] Headache [13 (2.9)] Sensitivity disorders [9 (2.0)] Movement disorders [5 (1.1)] Confusion, memory loss [16 (3.5)]	3–4 months after discharge, consulted by telephone
Rass et al. (2022)	Prospective study	Austria	*N*: 135 *N*: 81	M M	Hyposmia/anosmia, SS-16 < 13 [57 (45)] Cognition impairment MoCA (< 26) [29 (23)] Neuropathy/myopathy [16 (12)] Muscular debility acquired in ICU [8 (6)] Symmetric axonal distal neuropathy [7 (5)] Compression neuropathy [3 (2)] GBS [1 (1)] Hyposmia/anosmia, SS-16 < 13 [41 (51)] Cognition impairment MoCA (< 26) [14 (18)] Neuropathy/myopathy [8 (9)] Muscular debility acquired in ICU [1 (1)] Symmetric axonal distal neuropathy [3 (4)] Compression neuropathy [3 (4)]	Two follow-up visits (3-month and 1-year), neurological examination and a standardized test battery including the assessment of hyposmia (16-item Sniffin' Sticks test), cognitive deficits (Montreal Cognitive Assessment < 26)
Bungenberg and Humkamp (2022)	Cross-sectional study	Germany	*N*: 21	H	Fatigue [ 13 (60%)] Cognitive problems [18 (86)] Altered smell/taste [12 (57)] Paresthesia [2 (10)] Sensory deficit [9 (43)] Impaired fine motor skills [4 (19)] Paresis [3 (14)] CIP/CIM [9 (43)] Seizures [1 (5)] Stroke/TIA [2 (10)]	The median timespan after infection was 41 weeks (range 18.14–52.29), neurological examination, common standardized neuropsychological testing battery inter alia MoCA, TAP (Test of Attentional Performance), patient-reported outcome measures (PROMs) and MRI
Ali et al. (2022)	Prospective study	USA	*N*: 27	NH	Fatigue [22 (81)] Brain fog [16 (59)] Numbness/tingling [14 (52)] Headache [13 (48)] Dysgeusia [7 (26)] Anosmia [9 (33)] Dizziness [11 (41)] Blurred vision [8 (30)]	6–9 months after their initial Neuro-COVID-19 clinic evaluation. Phone/email questionnaire
Wong-Chew et al. (2022)	Prospective study	Mexico	*N*: 928	H	Fatigue [232 (25)] Headache [158 (17)] Lack of concentration [91 (9.8)] Loss of memory [78 (8.4)] Bradyphrenia [46 (5)] Disorientation [20 (2.1)] Paresthesia [97 (10.5)] Anosmia [32 (3.4)] Dysgeusia [25 (2.7)] Dizziness [60 (6.5)] Slow walking [37 (4)]	Over 90 days post-discharge, self-reported clinical symptom *via* telephone calls

* Hospitalized.

** Non-hospitalized.

*** Mixed (H + NH).

### Characteristic of neurological issues

**Figure 2:**
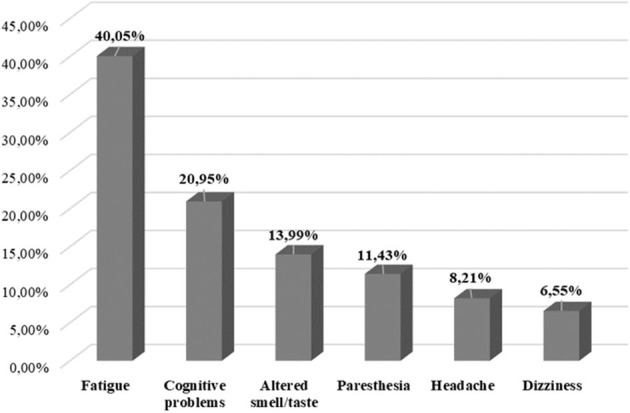
Percentage distribution of common neurological symptoms as late sequelae of SARS-CoV-2 infection.

In our study, “the most frequent” neurological consequences were fatigue and cognitive problems. Paresthesia and altered smell/taste were classified as “more frequent” and headache and dizziness were identified as “frequent” neurological symptoms in patients with COVID-19. Myalgia and blurred vision were identified as “possible significant neurological consequences” (Table 2). Figure 2 represents the percentage distribution of neurological symptoms as late sequelae of SARS-CoV-2 infection.

**Table 2:** Summary of neurological issues reported in patients as late sequelae of SARS-CoV-2 infection.

**Neurological issue**	**Total number studies**	**Total number of patients studied (*N*)**	**Number of patients showing symptoms (*n*)**	***n*/*N* (%)**	
Fatigue	15	6,444	2,581	40.05%	“The most frequent”
Cognitive problems	28	6,873	1,440	20.95%	
Altered smell/taste	21	7,484	1,047	13.93%	“More frequent”
Paresthesia	11	2,844	325	11.43%	
Headache	17	8,427	692	8.21%	“Frequent”
Dizziness	8	5,447	357	6.55%	
Myalgia	4	497	185	37.22%	“Possible significant neurological consequences”
Blurred vision	3	373	71	19.03%	
Vertigo	3	1,040	42	4.04%	“Other rare neurological consequences”
Movement disturbance	5	2,364	90	3.81%	
Muscular debility acquired in ICU	4	983	49	4.98%	
GBS	4	4	4	100.00%	
Seizures/status epilepticus	5	124	5	4.03%	
Sensory disfunction	2	71	12	16.90%	
Cranial nerve disfunction	1	50	5	10.00%	
Dysarthria	1	50	1	2.00%	
Dysphagia	1	50	1	2.00%	
Back/limb pain	2	200	64	32.00%	
Stroke	2	179	3	1.68%	
Bradyphrenia	1	928	46	4.96%	
Restless legs syndrome variant	1	1	1	100.00%	
Transfers myelitis	1	1	1	100.00%	
Functional movement disorders	1	1	1	100.00%	
Extended neuralgic amyotrophy syndrome	1	1	1	100.00%	

## Discussion

Our review presents the neurological issues that may be late consequences of SARS-CoV-2 infection. This article provides relevant information from 45 studies involving 9,746 patients. Pooled evidence showed that fatigue, cognitive problems, altered smell/taste, and paresthesia were very common neurological issues that were identified at least 4 weeks after a positive SARS-CoV-2 polymerase chain reaction (PCR) test and/or symptomatic start of confirmed SARS-CoV-2 infection. Other common neurological issues were headaches and dizziness.

Many studies reported a high rate of post-COVID-19 fatigue (Huang et al., 2020; Townsend et al., 2020; Wang et al., 2020). Fatigue is a non-specific symptom that accompanies many diseases, including infectious diseases. Of more than 6,000 studied patients from 15 different articles, more than 40% reported fatigue. It is worth mentioning that fatigue is reported in 5–45% of the general healthy population (Finsterer and Mahjoub, 2014). However, the studies included in our review did not focus on fatigue as the leading symptom. These studies evaluated various sets of symptoms with a neurological profile that accompanies the patient even for months after recovery from COVID-19. Bearing in mind the above data, it is difficult to conclude whether the problem of fatigue has increased during the pandemic. Therefore, further analysis of this issue is necessary.

There is a growing concern about the cognitive aspect of people who have recovered from COVID-19. Hampshire et al., based on cross-sectional cognitive performance data from 81,337 participants, observed that cognitive impairments were most pronounced in people who had been hospitalized but, importantly, were also observed in non-hospitalized patients with no reported breathing difficulties (Hampshire et al., 2021). Therefore, it is easier to understand the cognitive problems in patients hospitalized for COVID-19, who were more likely to have hypoxia, as well as septic complications. Hypoxia is a common cause of neuropsychological changes observed in acute respiratory distress syndrome (Hopkins et al., 2006). Patients who have required ventilation for multiple reasons may need help with daily tasks due to problems with attention, memory, verbal fluency, and information processing speed (Mikkelsen et al., 2012; Sasannejad et al., 2019). Thus, it is well-known that hypoxia, sepsis, and the accompanying immune hyperstimulation contribute to cognitive deficits.

It is less clear that patients with mild COVID-19 course, who have not been hospitalized, may also have objectively measurable Alzheimer's disease (AD)-like cognitive impairment (Albu et al., 2021b; Boesl et al., 2021; Papri et al., 2021). Although the specific mechanisms remain largely unknown, a recent study based on the use of single nuclear RNA sequencing datasets revealed associations between the pathogenic mechanisms of COVID-19 and AD. Researchers have identified significant similarities in neuronal damage, synaptic dysfunction, and neuroinflammation in both diseases. They presented the role of neural cell adhesion molecule 2 (NCAM2) and ICA1L (AD gene marker) in the process leading to cognitive impairment, which may be a potential target for AD intervention (Fu et al., 2022).

There is still too little information in the literature about baseline (before COVID-19 infection) clinical measures of cognitive-affective alteration. Therefore, it can only be assumed that the cognitive impairment may be either the result of the direct negative effects of the SARS-CoV-2 virus or the acceleration and aggravation of pre-existing cognitive deficits. Hence, advancing medical scientific knowledge through full case reports with included pre-COVID-19 status seems the most appropriate way. A case report of a young “33-year-old woman” with cognitive deficits 149 days after the first COVID-19 symptoms is a good example, as we can find a comparison for cognitive tests from 12 years ago (Hellmuth et al., 2021).

Cognitive decline is often undiagnosed until it is more advanced, leading to impairment of the ability to perform daily activities. The SARS-CoV-2 infection has spread to all continents, affecting particularly hard older people with comorbidities. This group of people often experiences a diminished quality of life resulting from new impairments with accompanying limitations in activities and restrictions to their participation in life. Therefore, it is necessary to focus on the possible cognitive impact of SARS-CoV-2 infection. When analyzing the topic of cognitive problems after SARS-CoV-2 infection, it is worth paying attention to the promising reports on the reversibility of cognitive disorders. Blazhenets et al. (2021) demonstrated essential reversibility of decreased neocortical glucose metabolism assessed by 18F-FDG PET accompanied by an improvement in cognitive functions in patients with COVID-19 (subjective and objective MoCa examination) from the subacute stage to the chronic stage after SARS-CoV-2 infection.

Future work would benefit from systematic cognitive assessments of ambulatory patients with COVID-19.

A complete or partial loss of smell and taste sensations is the most frequent neurological manifestation of COVID-19. Their occurrence can be explained as the expression of SARS-CoV-2 entry receptors in the olfactory epithelium. Then, SARS-CoV-2 *via* the olfactory nerve can spread to the olfactory bulb (Desforges et al., 2019; Beltrán-Corbellini et al., 2020). Clinicians should be alert regarding olfactory disorders which may mark the onset of some neurodegenerative diseases (Zhu et al., 2021).

Paresthesia is a common non-specific symptom with which patients come to the neurological clinic. There is evidence of changes in nociceptor excitability that COVID-19 could induce through multiple potential mechanisms (McFarland et al., 2021).

Other non-specific symptoms reported in the studies were headaches and dizziness. If they persist for several weeks, it is of concern among symptom-experienced people and physicians. This is often the reason for extended diagnostics procedures. During the COVID-19 pandemic, the incidence of headaches increased 5-fold in the studied region (Lippi et al., 2020). *De novo* headache is common post-COVID-19 and can persist long after infection resolution. Post-COVID-19 headache has often migraineurs features which may reflect an activation of the trigeminovascular system by inflammation or direct involvement of SARS-CoV-2. This hypothesis can be supported by concomitant anosmia (Caronna et al., 2020; Al-Hashel et al., 2021).

Moreover, Al-Hashel et al. (2021) found in the cohort study that a significant number of patients with primary headaches had worsening of their headaches within 3 months after COVID-19 disease. Headache and dizziness were presented very commonly in relevant studies included in our review.

The frequency was above 8 and 6% for headache and dizziness, respectively, reported in 17 different studies for headache and eight for dizziness.

It is worth keeping in mind that the COVID-19 pandemic may contribute to poor mental health manifested by somatization in the form of mental fatigue, cognitive changes, paresthesia, headaches, or dizziness.

Moreover, myalgia and blurred vision were identified as “possible significant neurological consequences”. Most cases of myalgia and blurred vision were self-reported and there was no information about the specificity of the symptoms. Rodriguez et al. (2021) considered whether myopathy is a part of long-COVID-19. They presented Multi Voltage Rule Check (MVRC) recordings 3 weeks after the onset of COVID-19 symptoms showed a marked reduction of early supernormality as a sign of muscle membrane depolarization compared to an earlier recording (Rodriguez et al., 2021).

In addition, it is worth referring to rare late neurological consequences of COVID-19 in our reviews, such as GBS and seizures. Most of the cases of GBS described in the literature are para- or directly postinfectious which is beyond the scope of our review. We found four relevant case reports related to GBS within the timeframe of our review where the interval between GBS and SARS-CoV-2 ranged from 1 month to 53 days. Keddie et al. (2021) compared GBS cases reported during the COVID-19 pandemic to GBS cases from 2016 to 2019. This epidemiological and cohort study investigated the UK population. Based on the comparison, the researchers concluded that GBS incidence has fallen during the pandemic. They assumed it might be caused by a lockdown that reduces transmission of GBS-inducing pathogens such as Campylobacter jejuni and respiratory viruses. There were no significant differences in the pattern of weakness, time to nadir, neurophysiology, CSF findings, or outcome between the COVID-19 pandemic group and the control groups (Keddie et al., 2021).

There are many descriptions of seizures during the acute infectious period in patients with COVID-19. Even convulsive and nonconvulsive status epilepticus triggered by SARS-CoV-2 virus infection has also been described (Emami et al., 2020; Somani et al., 2020; Asadi-Pooya et al., 2021). In our review, we focused on seizures/status epilepticus as a late consequence of COVID-19. Seizures are not a common late manifestation of COVID-19.

## Limitations

The main limitations were reliance on self-report measures in many articles. In some cases, there was a lack of clear information about comorbidities, so some symptoms may be due to pre-existing comorbidities. The same situation was about baseline assessment in the analyzed sources, which makes it impossible to reliably estimate the incidence.

Additionally, in some cases, the grouping of symptoms with an overlapping profile was used, which may have contributed to the fact that the frequency of some of the symptoms found in this review may be incorrectly estimated.

## Conclusion

According to data from World Health Organization (WHO) by 3 June 2022, the total number of COVID-19 cases worldwide reaches 528,816,317.00.

Assuming that only a minority percentage of patients with COVID-19 will struggle with late neurological issues when calculated on a global scale of patients affected with COVID-19, the prolonged neurological impact has become increasingly recognized and concerning.

We must remember that symptoms such as fatigue, cognitive problems, smell/taste disturbance, paresthesia or headache, and dizziness may accompany patients for many weeks after infection with SARS-CoV-2. Thus, recognition and familiarity with these neurological issues are imperative in managing these patients.

In our review, the long-term neurological consequences of COVID-19 disease have been collected and categorized in a simple and transparent way. Therefore, our study could be an easily accessible source of knowledge for medical professionals.

## Data availability statement

The original contributions presented in the study are included in the article/supplementary material, further inquiries can be directed to the corresponding author.

## Author contributions

KR: conceptualization, project administration, and supervision. AK: data curation, formal analysis, investigation, methodology, and writing—original draft. AK and KR: writing—review and editing. All authors contributed to the article and approved the submitted version.
